# Virtual reality stimulation to reduce the incidence of delirium in critically ill patients: study protocol for a randomized clinical trial

**DOI:** 10.1186/s13063-021-05090-2

**Published:** 2021-03-01

**Authors:** Aileen C. Naef, Marie-Madlen Jeitziner, Stephan M. Gerber, Béatrice Jenni-Moser, René M. Müri, Stephan M. Jakob, Tobias Nef, Matthias Hänggi

**Affiliations:** 1 Gerontechnology and Rehabilitation Group, University of Bern, Bern, Switzerland; 2 Department of Intensive Care Medicine, Inselspital, Bern University Hospital, University of Bern, Bern, Switzerland; 3 Department of Neurology, Inselspital, Bern University Hospital, University of Bern, Bern, Switzerland; 4 ARTORG Centre for Biomedical Engineering Research, University of Bern, Bern, Switzerland

**Keywords:** Delirium, Intensive care, Critical care, ICU, Virtual reality, Movement patterns, Randomized controlled trial

## Abstract

**Background:**

Delirium has been long considered as a major contributor to cognitive impairments and increased mortality following a critical illness. Pharmacologic and non-pharmacologic strategies are used against delirium in the intensive care unit (ICU), despite these strategies remaining controversial. Previous studies have shown the feasibility of using virtual reality within the ICU setting, and we propose to use this technology to investigate the effect of immersive virtual reality stimulation on the incidence of delirium in the ICU. Moreover, we propose to use motion sensors to determine if patient movement patterns can lead to early prediction of delirium onset.

**Methods:**

This study is conducted as a randomized clinical trial. A total of 920 critically ill patients in the ICU will participate. The control group will receive standard ICU care, whereas the intervention group will, in addition to the standard ICU care, receive relaxing 360-degree immersive virtual reality content played inside a head-mounted display with noise-cancelling headphones, three times a day. The first 100 patients, regardless of their group, will additionally have their movement patterns recorded using wearable and ambient sensors. Follow-up measurements will take place 6 months after discharge from the ICU.

**Discussion:**

Delirium is widely present within the ICU setting but lacks validated prevention and treatment strategies. By providing patients with virtual reality stimulation presented inside a head-mounted display and noise-cancelling headphones, participants may be isolated from disturbances on an ICU. It is believed that by doing so, the incidence of delirium will be decrease among these patients. Moreover, identifying movement patterns associated with delirium would allow for early detection and intervention, which may further improve long-term negative outcomes associated with delirium during critical care.

**Trial registration:**

ClinicalTrials.gov NCT04498585. Registered on August 3, 2020

## Administrative information

The order of the items has been modified to group similar items (see http://www.equator-network.org/reporting-guidelines/spirit-2013-statement-defining-standard-protocol-items-for-clinical-trials/).

**Table:** 

Title {1}	Virtual reality stimulation to reduce the incidence of delirium in critically ill patients: study protocol for a randomized clinical trial
Trial registration {2a and 2b}.	ClinicalTrials.gov, NCT04498585. Registered on August 3rd, 2020
Protocol version {3}	Version 2.0 5.02.2020
Funding {4}	Funding provided by the University of Bern, Switzerland, the Gerontechnology and Rehabilitation Group at the ARTORG Centre, and the Department of Intensive Care Medicine, Inselspital, Bern University Hospital, Switzerland.
Author details {5a}	Aileen Naef^1^, Marie-Madlen Jeitziner^2^, Stephan M. Gerber^1^, Béatrice Jenni-Moser^2^, René M. Müri^1,3^, Stephan M. Jakob^2^, Tobias Nef^3,4^, Matthias Hänggi^2^ ^1.^ Gerontechnology and Rehabilitation Group, University of Bern, Bern, Switzerland ^2.^ Department of Intensive Care Medicine, Inselspital, Bern University Hospital, University of Bern, Switzerland ^3.^ Department of Neurology, Inselspital, Bern University Hospital, University of Bern, Switzerland ^4.^ ARTORG Centre for Biomedical Engineering Research, University of Bern, Bern, Switzerland
Name and contact information for the trial sponsor {5b}	Prof. Dr. med. Stephan M. Jakob, Chief Physician, Department of Intensive Care Medicine, Inselspital, Bern University Hospital, University of Bern, CH-3010 Bern Tel.: + 41 (0)31632 39 38 / Email: stephan.jakob@insel.ch
Role of sponsor {5c}	The sponsor was involved in the study design, writing of the report, and the decision to submit the report for publication. The sponsor will also be involved in the interpretation of the data. The sponsor will not be involved in the collection, management, and analysis of the data.

## Introduction

### Background and rationale {6a}

Critically ill patients in the intensive care unit (ICU) frequently develop delirium. The prevalence of delirium in the ICU is between 35 and 80% in ventilated and non-ventilated patients [1, 2]. Delirium is defined as a disturbance in attention, awareness, and cognition with reduced ability to direct, focus, sustain, and shift attention, and reduced orientation to the environment [3, 4]. It profoundly affects both the patients and their families as it is associated with increased mortality [1, 5, 6], increased cognitive impairment [1, 5], longer duration of mechanical ventilation [1, 6, 7], unplanned extubation and catheter removal by the patient [6, 7], and longer ICU and hospital stay [1, 5–7]. Furthermore, it has been shown that the duration of delirium is associated with worse long-term cognitive impairment [8]. Delirium may occur as a hypoactive, hyperactive, or mixed form. Unfortunately, delirium often evades diagnosis as it most frequently presents with hypoactivity and somnolence [3]. Conversely, it can be more easily recognized when patients are hyperactive or agitated [3].

In prevention and treatment, pharmacological and non-pharmacological strategies are discussed [5]. Pharmacologic prevention and treatment of delirium remains controversial [5, 9, 10]. Despite this, in hyperactive patients, pharmacological measures are used in the clinical setting, with typical and atypical antipsychotics and dexmedetomidine recommended for controlling the symptoms [9]. In the Clinical Practice Guidelines for adult patients in the ICU, Devlin et al. do not recommend the use of haloperidol, atypical antipsychotics, or statins, for treating delirium except in patients experiencing distress, or showing signs of agitation or hallucinations, unrelated to the delirium [5]. All three drugs were not found to decrease delirium duration, duration of mechanical ventilation, ICU length of stay, or mortality [5]. Similarly to haloperidol, the effectiveness of dexmedetomidine in patients with delirium without agitation, or with agitation not related to being mechanically ventilated, remains unclear [5].

Despite non-pharmacological strategies also lacking clinical evidence of their efficacy, single- or multicomponent interventions, such as early mobilization, sleep improvement, and reorientation, are often used for preventing or treating delirium [5, 11]. One advantage of using multicomponent strategies, as opposed to single component approaches, proposed in the literature, is that multicomponent strategies may target more than one risk factor for delirium simultaneously [12]. In this way, there is a greater potential of having a beneficial effect, even in the absence of strict compliance.

In a pilot study, Gerber et al. [13] showed the feasibility, usability, and acceptance of virtual reality (VR) stimulation (i.e. relaxing nature videos) as a new non-pharmacological intervention to comfort patients during their stay in the ICU. Currently, head-mounted displays have been widely used therapeutically in individuals with various mental and physical disorders [14–16]. Two studies conducted by Gerber et al. [17, 18] produced evidence that VR stimulation with a head-mounted display in ICU settings is potentially beneficial for patients. More specifically, the relaxing effect produced by the VR stimulation has a high potential to reduce the incidence of delirium. Furthermore, VR-based cognitive stimulation in critically ill patients did not evoke any negative reactions and is thus safe to use in this population [13, 17, 18].

For the two main aims of this randomized clinical trial, we focus on (1) reducing the incidence of delirium by using VR stimulation and (2) analysing movement patterns in order to obtain indicators as to the occurrence of delirium.

### Objectives {7}

The primary objective of this randomized clinical trial is to analyse the effect of VR stimulation on the incidence of delirium after admission to the ICU. Participants in the control group will receive the standard ICU care, while participants in the intervention group will additionally receive VR stimulation. We hypothesize that participants receiving the VR stimulation will have a lower incidence of delirium during their stay in the ICU, compared to the control group. The secondary objectives are to evaluate differences in movement patterns and intensity between the intervention and control groups and between participants with delirium and without delirium. We expect that these differences will be related to the different types of delirium (i.e. hyperactive, hypoactive, or mixed). Additionally, it is predicted that it will be possible to detect differences in movement patterns between groups and identify patterns indicating the presence or absence of delirium. Secondary objectives also include the evaluation of the effect of VR on duration of delirium. In this case, it is hypothesized that the duration of delirium will be shorter in participants receiving the VR stimulation compared to those in the control group.

Further objectives which will be addressed include (1) evaluating the effect of VR stimulation, identified by changes in physiological parameters; (2) evaluating how easily the VR stimulation can be provided to patients by critical care professionals; (3) describing differences in cognition, health-related quality of life, and functional independence between groups, using questionnaires; and (4) evaluating if VR reduces the need for pharmacological treatment of delirium.

### Trial design {8}

This study is conducted as a randomized clinical trial with a superiority framework and a parallel group design. There are two distinct groups; the control group will receive standard ICU care, whereas the intervention group will, in addition to the standard ICU care, receive relaxing VR stimulation three times a day.

## Methods: participants, interventions, and outcomes

### Study setting {9}

The study will be conducted in the interdisciplinary adult ICU of the University Hospital of Bern, (Inselspital), a Swiss university hospital. Data will be recorded solely at this location.

### Eligibility criteria {10}

All patients in the ICU, including patients with acquired brain injury, are eligible to participate in this study. The interventions will be performed by trained ICU nurses.

#### Inclusion criteria

- Aged ≥ 18 years with no upper age limit
- No severe visual or auditory impairments (diplopia, low vision due to macular degeneration, retinopathy, severe hypacusis, or deafness)
- Estimated length of stay > 24 h
- Can keep eyes open for at least 30 s
- German or French speaking

#### Exclusion criteria

- Known psychotic disorders associated with delusions (e.g. schizophrenia, dementia)
- Recent history of major depression
- Admission for drug overdose

### Who will take informed consent? {26a}

All patients who meet the inclusion criteria will be approached by a member of the study team within 24 h of ICU admission. However, since patients are often unable to give consent due to their condition and the situation of their relatives or authorized representative is often unclear at the beginning, a neutral physician may be consulted before the patient is included in the study. The neutral physician who is not involved in the study and who safeguards the participant interests provides a written authorization to enrol the patient.

If a proxy consent is not obtainable because no relatives and/or representative is identified or the representative cannot be reasonably contacted, the patient will stay in the study and their data will be used in order not to compromise the results of the study.

In the case where the patient is discharged from the ICU in a state of capacity that does not allow him/her to provide consent, a re-evaluation of their capacity to consent will only be done during the study follow-up visits or telephone follow-up calls (6 months visits). If the patient cannot provide informed consent at 6 months post-discharge, the relatives and/or representative will be asked again to provide informed consent to use the study data.

Informed consent will be obtained by a member of the study team after providing the patient, family member(s), or authorized representative with both verbal and written study information about what the study entails (e.g. its benefits, risks, design) and what the informed consent states. If the patient, family member(s), or an authorized representative agrees to participate in the study, they will be asked to sign a written consent form for their study participation. If a family member or authorized representative provides consent on the patients’ behalf, the patient will be asked directly for their consent as soon as their health condition allows. The patient will again be asked for their written informed consent 6 months post-ICU discharge to participate in a 6-month follow-up visit.

### Additional consent provisions for collection and use of participant data and biological specimens {26b}

Not applicable, all data which will be collected from the participant is outlined in the informed consent and would only be used to address the topics listed among the objectives of this study. Moreover, this trial does not involve collecting biological specimens for storage.

## Interventions

### Explanation for the choice of comparators {6b}

In this study, we have chosen to use a control group as a comparator. The control group is made up of individuals who are also patients hospitalized in the ICU and receive the same standard ICU care to address their health needs as participants in the intervention group, only without the VR stimulation.

### Intervention description {11a}

The intervention for this study consists of relaxing VR stimulation, which will be started as soon as possible after obtaining informed consent. As stimulation material, immersive 360-degree videos (e.g. nature environments, animals, urban parks) will be played inside a commercially available head-mounted display, with the video sound played inside noise-cancelling headphones. A single video will be shown during each stimulation period, with each video lasting 30 min in length; however, the length may be shortened based on individual participant reaction. The stimulation will be provided three times per day, morning, midday, and evening, every day until the patient is discharged from the ICU, or the maximum of 14 days has been reached. A new video will be played during each stimulation. A total of 44 videos were filmed. All videos shown on the same day will depict the same location, but at different time points. The location shown on a given day is randomly assigned. Videos filmed in the morning, midday, or evening will be shown in the morning, midday, or evening, respectively.

The intervention group will also receive standard ICU care and, if needed, receive treatment for delirium (local delirium protocol), same as in the control group. Therapy would include pharmacological agents (e.g. quetiapin) and non-pharmacological interventions (e.g. early mobilization, communication tools, sleep improvement, family involvement). The first 100 participants, regardless of their group, will additionally be equipped so data can be recorded regarding movement patterns.

### Criteria for discontinuing or modifying allocated interventions {11b}

Participants will be withdrawn from the study if they voluntarily withdraw informed consent at any time. Moreover, participation in the study will be discontinued if:

- There are changes in their health status that makes continued participation inadvisable
- There are safety concerns

The target length of the intervention is 30 min, but stimulation can be stopped prior to achieving this goal per participant request, or perceived negative reaction of the participant, regardless of the discontinuation criteria listed above.

### Strategies to improve adherence to interventions {11c}

VR stimulation will be provided to the participants by trained ICU nurses who are part of the study team; therefore, adherence while in the ICU should be very high. To ensure adherence at 6 months post-ICU discharge, the participants will be contacted by a member of the study team. The follow-up visit would also take place during a standard patient follow-up appointment.

### Relevant concomitant care permitted or prohibited during the trial {11d}

All standard ICU care to treat the participant during their time in the ICU will be permitted.

### Provisions for post-trial care {30}

In the event of study-related damage or injuries, University Hospital Bern (Inselspital) shall provide compensation, except for claims that arise from misconduct or gross negligence of involved study personnel.

### Outcomes {12}

The primary outcome is delirium incidence, measured three times a day (morning, midday, and evening) by the Intensive Care Delirium Screening Checklist (ICDSC) as part of standard assessment. With the ICDSC, delirium is determined by consciousness, inattention, disorientation, hallucination, agitation or retardation, speech and mood, sleep/wake cycle disturbance, and fluctuation [19]. A unique incidence of delirium is counted once a patient reaches a score of ≥ 4, on a scale from 0 to 8 [20]. The entire period that a patient has a score ≥ 4 is considered as a single episode, and accordingly, counts only as a single incidence. The mean incidences of delirium per group will be looked at. The incidence of delirium is considered to be clinically relevant as it is known that patients who experience delirium while in critical care have worse long-term outcomes [4]. Furthermore, due to the possible influence of sedatives and analgesics on the incidence of delirium, both sedation and pain levels will be assessed every 2 h [5]. Consciousness and sedation will be measured using the Glasgow Coma Scale [21] and the Richmond Agitation Sedation Scale [22], respectively, and pain will be measured using the Numeric Rating Scale [23] and the Behavioural Pain Observation Tool [24].

As a secondary outcome, the duration of delirium will be evaluated per 8-h periods, or as needed, while patients are in the ICU. If the participant is assessed as delirious via the ICDSC during any of the assessments during an 8-h span, the entire period will count as delirious. Duration of delirium is clinically relevant knowing that the duration of delirium is an independent predictor of mortality 6 and 12 months post-ICU admission [25, 26].

Additional secondary outcomes include the pattern and intensity of movements, before and during delirium as well as between groups, measured continuously during the ICU stay. The fact that patients in delirium have fluctuating motor performance movement patterns, differing from baseline, could be a possible marker for delirium detection [27]. Therefore, acceleration will be measured with wearable sensors such as inertial measurement units, which have been previously used successfully in the ICU [28]. The monitoring of patients’ movement patterns could provide a means of early detection of delirium, permitting early intervention by the care staff.

Other pre-specified outcome variables include physiological parameters, feasibility of using the VR stimulation by ICU nurses, cognition at ICU discharge and 6 months post-discharge, health-related quality of life and functional independence before ICU admission and 6 months post-discharge, disease-related data, and survival 6 months post-discharge from the ICU. Physiological parameters (e.g. blood pressure, heart rate, respiration rate) will be recorded continuously and changes from baseline, or the start of an event such as VR stimulation, will be compared to later time points. The changes occurring in these parameters over time, together with disease-related data, are of interest and could indicate what effect, if any, VR stimulation has on the condition of the participant. Scores for health-related quality of life, functional independence, and clinical frailty before ICU admission and 6 months post-discharge, and cognition before ICU discharge and 6 months post-discharge, will be compared to determine if any significant differences occur. Additionally, the average survival 6 months post-ICU discharge will be compared between groups. Looking at these variables between groups is important as cognition, health-related quality of life, independence, frailty, and mortality, among others, are known to be negatively impacted by the presence of delirium during the time spent in the ICU. Therefore, it is meaningful to identify if there were any significant changes in these aspects for individuals in the treatment group compared to the controls. It will also be important to determine if providing such an intervention is feasible for the care providers. For this, the validated System Usability Scale will be completed by the nurses in order to understand if overall, the use of this technology was accepted and whether or not it could be integrated into standard ICU care practices [29].

### Participant timeline {13}

**Fig. 1:**
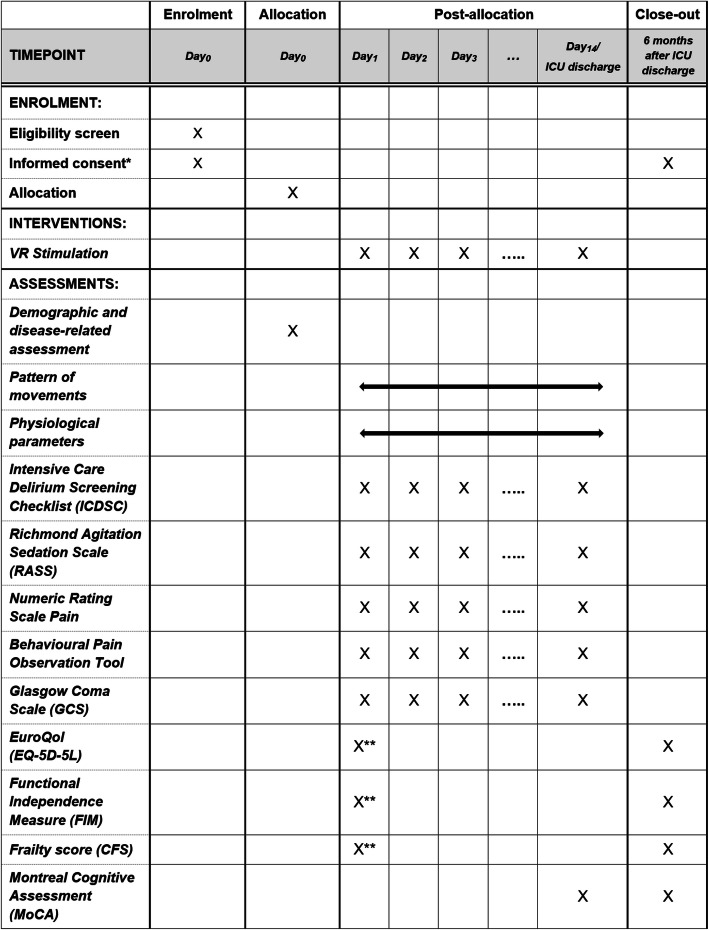
Schedule of enrolment, intervention, and assessments. Arrows represent continuous measurements, and X’s represent an assessment or intervention provided at a specific time point. One asterisk indicates that whenever possible, informed consent will be obtained directly from the patient. If the patient is unable to provide informed consent, we will ask the patients’ relatives and/or an authorized representative for the study consent. Two asterisks indicate that we will ask the patients’ relatives at the earliest possible date, with questions regarding the week prior to arrival at the ICU

Participant recruitment, enrolment, and allocation will all be targeted for the first 24 h following admission to the ICU. Once admitted, the participants will be monitored and evaluated, through a series of assessments, every day they are in the ICU up until their discharge, or at most, 14 days following ICU admission. A follow-up visit is planned for 6 months post-ICU discharge (Fig. 1).

### Sample size {14}

The sample size calculation is based on testing of the primary outcome. Based on trials conducted by Boettger et al. [30] and Bounds et al. [31], we assume that 39% and 30% of participants will have delirium in the control and intervention groups, respectively. A total sample size of 920 participants (460 per group) provides a power of 80% to detect a significant improvement, and thereby a decrease, in the proportion of delirium with a two-sided significance threshold of 0.05. The statistical power to reject the null hypothesis under the above assumptions is 0.95 and the overall type I error rate is 0.05. However, the prevalence of delirium in the ICU being reported is between 35 and 80% making it difficult to calculate an exact power. We will, therefore, conduct a blinded interim analysis after inclusion of 100 participants (50 per group); re-estimation and adjustment of the sample size will be made if necessary.

### Recruitment {15}

With approximately 4000 patients [32] admitted yearly to the ICU at the University Hospital of Bern (Inselspital), we believe achieving our total sample size of 920 participants over the 3 years for which our study is planned is feasible. All patients admitted to the ICU will be screened for inclusion into the study, by either a registered nurse or a treating physician. Family members will be contacted as soon as possible thereafter. Patients will be recruited every day, including on the weekends.

## Assignment of interventions: allocation

### Sequence generation {16a}

In the enrolment phase, ICU patients will be randomly assigned to either the intervention or the control group with 1:1 allocation, stratified by gender and age. The randomization will be computer-generated and performed in the study database using the Research Electronic Data Capture System (REDCap) (Vanderbilt University, TN, USA). The research team will not be blinded.

### Concealment mechanism {16b}

The allocation sequence will be implemented in the study database REDCap by an independent data manager not involved in the analysis of the study. No other study personnel will have access to the allocation sequence that is stored in REDCap. Allocation of individual patients to their respective intervention group will be revealed to study personnel after the allocation has occurred via the web interface of REDCap.

### Implementation {16c}

The allocation sequence assigning participants to their respective group will be computer-generated. The study team will enrol participants prior to allocation.

## Assignment of interventions: blinding

### Who will be blinded {17a}

The research team will not be blinded after assignment to interventions, nor will any member of the interprofessional team be blinded when carrying out the intervention. However, an independent statistician who will be conducting the interim analysis will be blinded.

Additionally, the analysis of the primary outcome of incidence of delirium, and secondary outcome looking at the duration of delirium, will be done blinded.

### Procedure for unblinding if needed {17b}

Only one person, the independent statistician, is blinded during the intervention period of this study. If required, unblinding could occur after the interim analysis has been conducted.

## Data collection and management

### Plans for assessment and collection of outcomes {18a}

The study starts within 24 h after admission to the ICU. In the enrolment phase, participants will be randomly assigned to either the intervention or the control group, with the intervention beginning as soon as possible following enrolment. Three times a day (between 7:00 am and 7:00 pm) for 30 min, participants in the intervention group will receive the intervention (VR stimulation) applied directly in the bed. The intervention will be included in the daily schedule of the patients. The intervention period ends when the patient is discharged from the ICU or at the latest 14 days after admission to the ICU. Thus, the intervention period depends on the length of stay in the ICU and can vary between participants.

After allocation, incidence and duration of delirium is assessed in three shifts a day. If delirium is diagnosed, the standard ICU delirium therapy (local delirium protocol) will be started, regardless of the participants’ group. VR stimulation will optimally start before this time point. Delirium is assessed using the ICDSC which is a reliable (Cohen’s kappa (*κ*) = 0.60) [33] and specific (95%) [33] tool to detect delirium in ICU patients [5] and was translated into German by Radtke et al. [34]

Assessment of sedation will be done using the Richmond Agitation Sedation Scale which has a reliability of *κ* = 0.91 and a face validity with 81–92% agreement [22, 35]. Consciousness will be evaluated according to the Glasgow Coma Score which was originally found to have a *κ* = 0.66–0.77 for the whole scale, but has since been found to have higher reliability when looking at the eye, motor, and verbal scales individually (*κ* = 0.89, 0.94, and 0.88, respectively) [21, 36]. The validity for the Glasgow Coma Scale, however, remains controversial with some stating that the validity has been well established [37], while others question how the scale is assessed under different conditions such as sedation or intubation [36]. Pain will be assessed using the Numeric Rating Scale with an interrater reliability score of *κ* = 0.71 [23] and the Behavioural Pain Observation Tool which has a *κ* = 0.80 and a content validity index of 0.73–1.0 [24].

Movement and movement patterns will be recorded continuously at 100 Hz for the duration of the participants’ stay in the ICU, with AX3 Axivity (Axivity Ltd., Newcastle upon Tyne, UK) inertial measurement units attached to participant’s hands, legs, and chest and placed under the mattress (Sensing Tex, Barcelona, Spain). To enable accurate interpretation of this data, the participant’s environment will be recorded with a camera. This will allow for the distinction to be made between movements made by the participant and those from the interprofessional team or family members. To maximize the privacy of the participant, the camera will be mounted on the head-end of the bed, so the face of the participant is not visible.

Physiological parameters (e.g. non-invasive arterial blood pressure, heart rate, and respiratory rate) will be monitored by the in-house monitor system of the University Hospital (GE Healthcare, Little Chalfont, UK). Respiration and heart rate will be measured at a frequency of 240 Hz, while the blood pressure will be measured as the median every 2 min. A list of pharmacological agents provided to the participant and disease-related data will be collected via the patient data management system. While movement and physiological data will be recorded continuously throughout the patients’ stay in the ICU, the non-continuous assessments will also be completed at various time points (Fig. 1).

At the earliest opportunity, the participant—or, in the event of incapacity, his/her relatives—will be interviewed by the study team about their health-related quality of life, functionality, and frailty, using the EQ-5D instrument [38], the Functional Independence Measure [39, 40], and the Clinical Frailty Score [41], respectively, 1 week before ICU admission and 6 months post-discharge. The reliability and validity of the three-level EQ-5D, the EQ-5D-3L, is well established; however, the more recently expanded five-level version, the EQ-5D-5L, still lacks proper reliability and validity studies [38]. The Functional Independence Measure has a reliability score of *κ* > 0.85 [42, 43] and a high internal validity [42]. The Clinical Frailty Score has been found to have good reliability [44] and good construct validity [41].

Cognitive impairment will be assessed by the study team before ICU discharge and 6 months post-discharge using the Montreal Cognitive Assessment [45]. The Montreal Cognitive Assessment has a known reliability of *κ* = 0.83 [45] and an established content validity in English and in French [45] and a sensitivity of at least 0.90 [46]. The average survival 6 months post-discharge will be obtained via a central database.

The study ends with a follow-up assessment 6 months after admission to the ICU. The study team interviews the participants during a regular follow-up session at the ICU. During the interview, the EQ-5D-5L, Functional Independence Measure, Clinical Frailty Score, and Montreal Cognitive Assessment will be administered. All assessments will be conducted according to the standard ICU care by trained ICU nurses.

In a final step following the VR intervention, the ICU nurse responsible for the intervention will be asked about the feasibility of applying the intervention, completing the 10-item System Usability Scale [29]. The System Usability Scale has a strong reliability score *κ* > 0.89 [47, 48] and an established construct validity, as well as concurrent validity (*r* = 0.86) [47, 48].

### Plans to promote participant retention and complete follow-up {18b}

Contact will be made with the participants after 3 months within the framework of internal quality assurance. After that, the participant will be contacted in writing and by telephone for the 6-month follow-up. If a participant chooses to discontinue the study, the data collected up to the withdrawal date will be anonymized and used.

### Data management {19}

Smaller datasets and measurements (e.g. length of ICU stay) will be stored in the patient data management system of the University Hospital of Bern (Inselspital). The data will be coded by a member of the study team using an alphanumeric system, and the identifying information deleted from the datasets. Only the remaining continuous data, such as physiological measurements, movement patterns, and camera videos, will be stored in-house on a password-protected computer due to their size.

### Confidentiality {27}

The data files are encrypted and will only contain a univocal alphanumeric code, from which the identity of the participant cannot be gathered. The key (i.e. the list in which a code number is linked to a participant name) will be kept separately from the study data, in a secured cabinet, in the custody of a person who is not involved in the study. In study-specific documents, participants are only identified by a unique participant number.

### Plans for collection, laboratory evaluation, and storage of biological specimens for genetic or molecular analysis in this trial/future use {33}

See above Item 26b, there will be no biological specimens collected.

## Statistical methods

### Statistical methods for primary and secondary outcomes {20a}

Descriptive statistics will be applied to both groups to ensure that they similarly represent the population. Among others, we will be looking at the mean and standard deviation of the age, distribution of the sexes, and frequencies of disease in the two samples.

The difference in the incidence of delirium will be tested with the chi-square proportion test, and the duration will be tested with Welch’s *t* test in subgroups of participants with delirium. The effects of the VR stimulation on physiological parameters will be looked at using statistical methods (e.g. *t* test, linear models). Machine learning techniques will be applied to the movement patterns as means of analysis.

The data will be analysed with MATLAB and the software R. Deviation(s) from the original statistical plan will be granted if the collected data do not allow the planned analysis techniques to be performed (e.g. if the collected data does not meet the prerequisites to apply analysis of variance techniques). In this case, more appropriate statistical methods will be applied (e.g. non-parametric methods), and this choice will be justified and detailed in the respective scientific reports.

### Interim analyses {21b}

A blinded interim analysis will be completed after the inclusion of 100 participants (50 per group). A re-estimation and adjustment of the sample size will be made if necessary. The decision about how to proceed following the results of the interim analysis will be made by the independent statistician conducting the interim analysis, together with the principal investigator.

### Methods for additional analyses (e.g. subgroup analyses) {20b}

Using data from movement patterns from a subgroup of participants (*n* = 100), non-supervised machine learning techniques will be applied to try to train a model to predict the onset of delirium. This subgroup will be used to address the secondary objective aimed to evaluate differences in movement patterns and intensity between the intervention and control group and between participants with delirium and without delirium.

### Methods in analysis to handle protocol non-adherence and any statistical methods to handle missing data {20c}

As suggested by the SPIRIT guidelines, we propose to test superiority using two analysis sets: the “intention-to-treat set”, considering all patients that are randomized regardless of whether they received the randomized treatment, and the “per protocol” set [49]. We propose declaring superiority of the experimental intervention only if shown superiority using both the “intention to treat” and the “per protocol” analysis set. As our study takes place in the ICU, it is expected that drop-outs will occur for medical reasons which make it unfeasible to continue in the study, but that are not related to the study itself. For this reason, there are two groups of participants who will be considered drop-outs. Firstly, any participant who can no longer continue in the study due to health reasons, before the first assessment for the primary outcome (i.e. the ICDSC) has been completed, will be considered a drop-out. Secondly, any participant for whom the neutral physician gave consent, but for whom the relatives withdrew consent, will be considered as a drop-out. Therefore, in order to maintain a sufficient sample size, it is important that these participants are replaced in a 1:1 manner, as approved by the local ethics committee. If a participant does drop-out of the study, their health status will be recorded at this point. As no long-term negative outcomes from the intervention are suspected, no further follow-up will be performed. If missing values occur within the dataset, we will use multiple imputation assuming missing data to be missing at random.

### Plans to give access to the full protocol, participant-level data, and statistical code {31c}

We will ask patients to provide informed consent for sharing their anonymized data. Once the study results as outlined in this proposal are published, we will make the de-identified study dataset available for secondary analyses by sharing the dataset upon reasonable request.

## Oversight and monitoring

### Composition of the coordinating centre and trial steering committee {5d}

The study will be monitored by an independent internal monitor. The monitor will evaluate the primary and secondary outcomes for completeness and correctness as outlined by the study protocol. Any decisions needing to be taken regarding the study will be done with the consensus of the entire study team and the relevant study authorities will be notified.

### Composition of the data monitoring committee, its role, and reporting structure {21a}

The study will be internally monitored according to the Good Clinical Practice directive and the monitoring and data verification plan, including the documentation of informed consent of study participants. Internally implies that the monitor is employed at the Department of Intensive Care Medicine, University Hospital of Bern (Inselspital), but carries out the monitoring independently, as they are neither involved in the study-specific processes (recruitment, study data collection, study interventions, or evaluation) nor in patient care. In their employment, they are administratively and functionally independent from the nursing and medical sector of the department.

### Adverse event reporting and harms {22}

In the scope of this study, no serious adverse events or adverse events are expected. Due to the serious condition of critically ill patients, adverse events will only be reported in the medical record if they are related to the VR intervention. Serious adverse events assessed with a causal relationship to VR will be reported to the ethics committee by the investigator and sponsor-investigator within 15 days.

### Frequency and plans for auditing trial conduct {23}

For quality assurance, the sponsor, the ethics committee, or an independent intern study monitor may visit the research site. Direct access to the source data and all study-related files is granted on such occasions. All involved parties will be required to keep the participant data strictly confidential.

### Plans for communicating important protocol amendments to relevant parties (e.g. trial participants, ethical committees) {25}

Substantial changes to the study setup and study organization, the protocol, and relevant study documents will be submitted to the ethics committee for approval before implementation. Under emergency circumstances, deviations from the protocol to protect the rights, safety, and well-being of human subjects may proceed without the ethics committee’s prior approval. Such deviations shall be documented and reported to the ethics committee as soon as possible. A list of all non-substantial amendments will be submitted once a year to the competent ethics committee together with the annual safety report.

### Dissemination plans {31a}

The results of the present study will be submitted for publication in peer-reviewed journals and will be presented at national and international scientific meetings. Currently, there are no known publication restrictions foreseen. The dataset will be available upon reasonable request once results have been published.

## Discussion

One challenge we will face during this study is the unpredictable reaction of patients to the VR stimulation when they are in a state of delirium. As such, the feasibility of providing VR stimulation to participants when they are already in a state of delirium is unknown. This can specifically be a problem due to their increased motor activity if they are in hyperactive delirium. Furthermore, there is a risk that delirious participants will not tolerate wearing such a head-mounted display and headphones and may even try to remove the items themselves. This may become a limitation in participant recruitment, as it does occur that patients enter into a state of delirium shortly after ICU admission. The study team will continue to provide the stimulation for as many days, and as consistently as possible; however, participant safety and comfort is the priority and, therefore, this aspect of unpredictability remains.

There currently remains the open question as to what specific aspect of the VR may be responsible for the effect we see, if any. Specifically, it is not known if it is the combination of audio and visual input, or the one or the other alone that creates an effect. Alternately, it could be that using these devices allows the patient to avoid noise overstimulation by using headphones and visual overstimulation by using the headset. This remains an open question.

It should be kept in mind that a limitation of the current study is the sparse literature confirming the reliability and validity of the questionnaires listed above, specifically for use in a language other than English and for our target group of ICU patients. The reliability and validity of the measures listed earlier are largely based on English speakers across a variety of patient populations and healthy controls. Therefore, we acknowledge that these values may not hold true, and applying it to participants in a language other than English may result in bias being introduced. Regarding the validity of the ICDSC, which we use for our primary outcome, the validity for the German version has not been assessed. Because of this, we will cross-check the score of the ICDSC with the Richmond Agitation Sedation Scale score. A positive Richmond Agitation Sedation Scale score is an important indicator of delirium based on symptoms that keep patients in the ICU, and therefore, should correspond to the ICDSC score.

## Trial status

Protocol version 2.0 (May 02, 2020)

Recruitment is expected to begin in March 2021 and continue until March 2024.

## Data Availability

The dataset will be available upon reasonable request once the results have been published.

## Abbreviations

REDCap: Research Electronic Data Capture System
ICDSC: Intensive Care Delirium Screening Checklist
ICU: Intensive care unit
VR: Virtual reality
